# Supplementary material for: Oxidized TRX1 and DPP8/9 stabilize the MIA40-AIFM1 complex during intrinsic apoptosis
Source: bioRxiv. 2026 Oct 28:2026.10.26.761918. Preprint. [Version 1] doi: 10.64898/2026.10.26.761918 (PMC13733872; doi:10.64898/2026.10.26.761918)
Supplement: Supplementary file 1 [file NIHPP2026.10.26.761918V1-supplement-1.pdf]

bioRxiv preprint doi: https://doi.org/10.64898/2026.10.26.761918; this version posted October 28, 2026. The copyright holder for this preprint (which was not certified by peer review) is the author/funder, who has granted bioRxiv a license to display the preprint in perpetuity. It is made available under a CC-BY 4.0 International license.

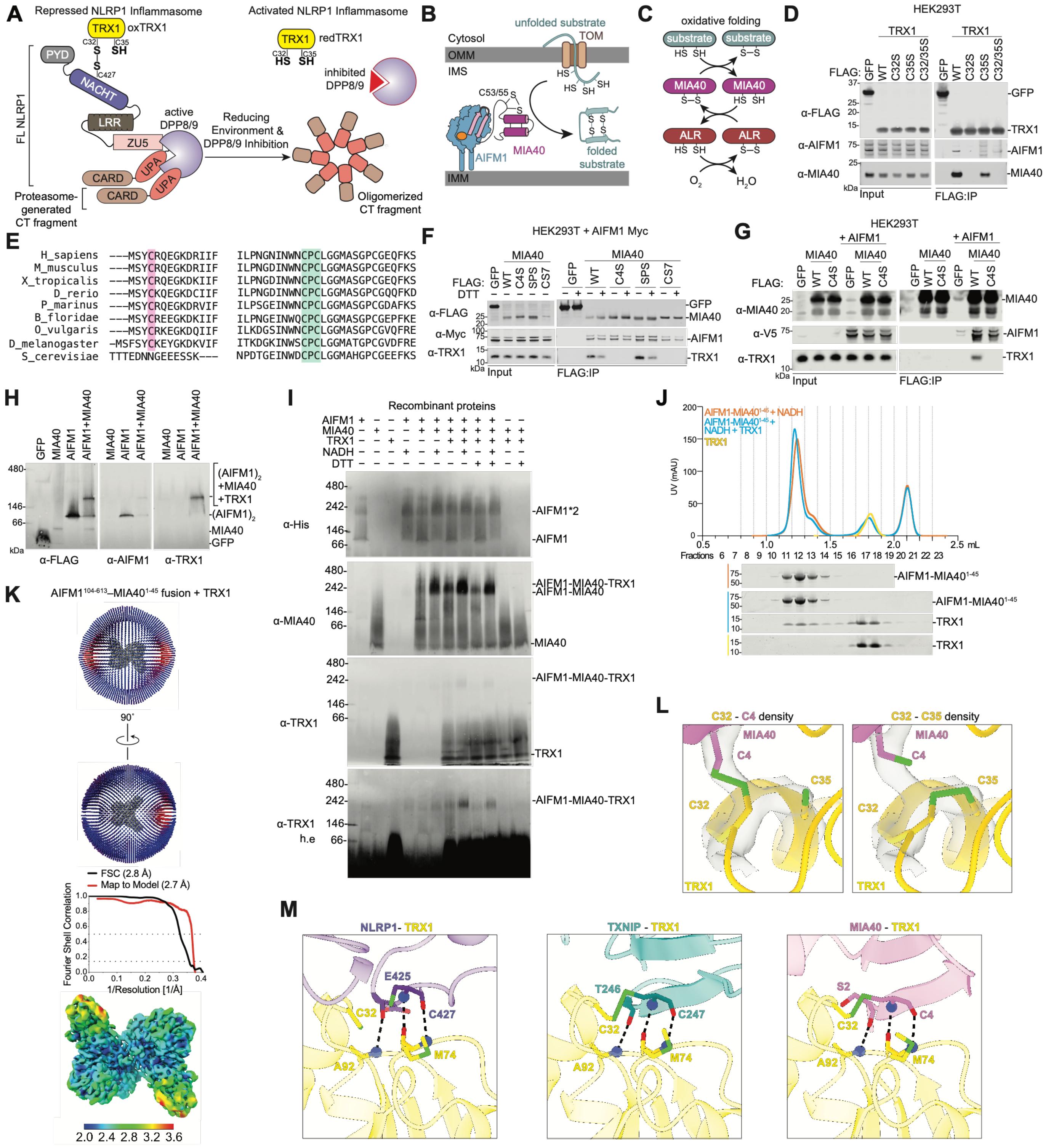

Figure S1. Characterization of the TRX1–AIFM1–MIA40 complex. **(A)** NLRP1 inflammasome regulation. NLRP1 autoproteolysis generates N-terminal (NT) and C-terminal (CT) fragments that remain noncovalently associated. oxTRX1 binding protects the NT fragment from degradation, whereas DPP8/9 bind FL NLRP1 together with the proteasome-released CT in a ternary complex that sequesters the CT. A reducing environment and DPP8/9 inhibition can relieve these restraints, leading to CT oligomerization and inflammasome activation. **(B–C)** MIA40-dependent mitochondrial protein import **(B)** and oxidative folding **(C)**. **(D,F)** The indicated proteins were expressed in HEK293T cells, IPed, and analyzed by immunoblotting. In **(F)** the beads were treated with or without DTT (50 mM) before immunoblotting. **(E)** Sequence alignment of MIA40 residues surrounding C4 and the CPC motif across the indicated species. **(G)** Immunoblots corresponding to **Figure 1G** samples. **(H,I)** BNP immunoblot analyses of the indicated protein complexes isolated from HEK 293T cells **(H)** or assembled using recombinant AIFM1, MIA40, and TRX1 (Cys 62/69/73 mutated to Ser) ± NADH (100 µM) and DTT (10 mM) (**I**). **(J)** Size-exclusion chromatography of the AIFM1–MIA40$^{1\text{-}45}$ fusion protein (50 µM) ± TRX1 (Cys62/69/73Ser, 120 µM) and NADH (100 µM). **(K)** Cryo-EM data processing and validation for the TRX1–MIA40–AIFM1 complex and local resolution estimate of the cryo-EM map. (**L**) Cryo-EM density corresponding to either an intermolecular MIA40 Cys4–TRX1 Cys32 disulfide (left) or an intramolecular TRX1 Cys32–Cys35 disulfide (right). **(M)** Structural comparison of the TRX1 interfaces with NLRP1, TXNIP, and MIA40 (dotted lines, hydrogen bonds), showing conserved backbone hydrogen bond interactions flanking their respective disulfide cysteine residues. Immunoblots and chromatograms are representative of three or more independent experiments.

bioRxiv preprint doi: https://doi.org/10.64898/2026.10.26.761918; this version posted October 28, 2026. The copyright holder for this preprint (which was not certified by peer review) is the author/funder, who has granted bioRxiv a license to display the preprint in perpetuity. It is made available under a CC-BY 4.0 International license.

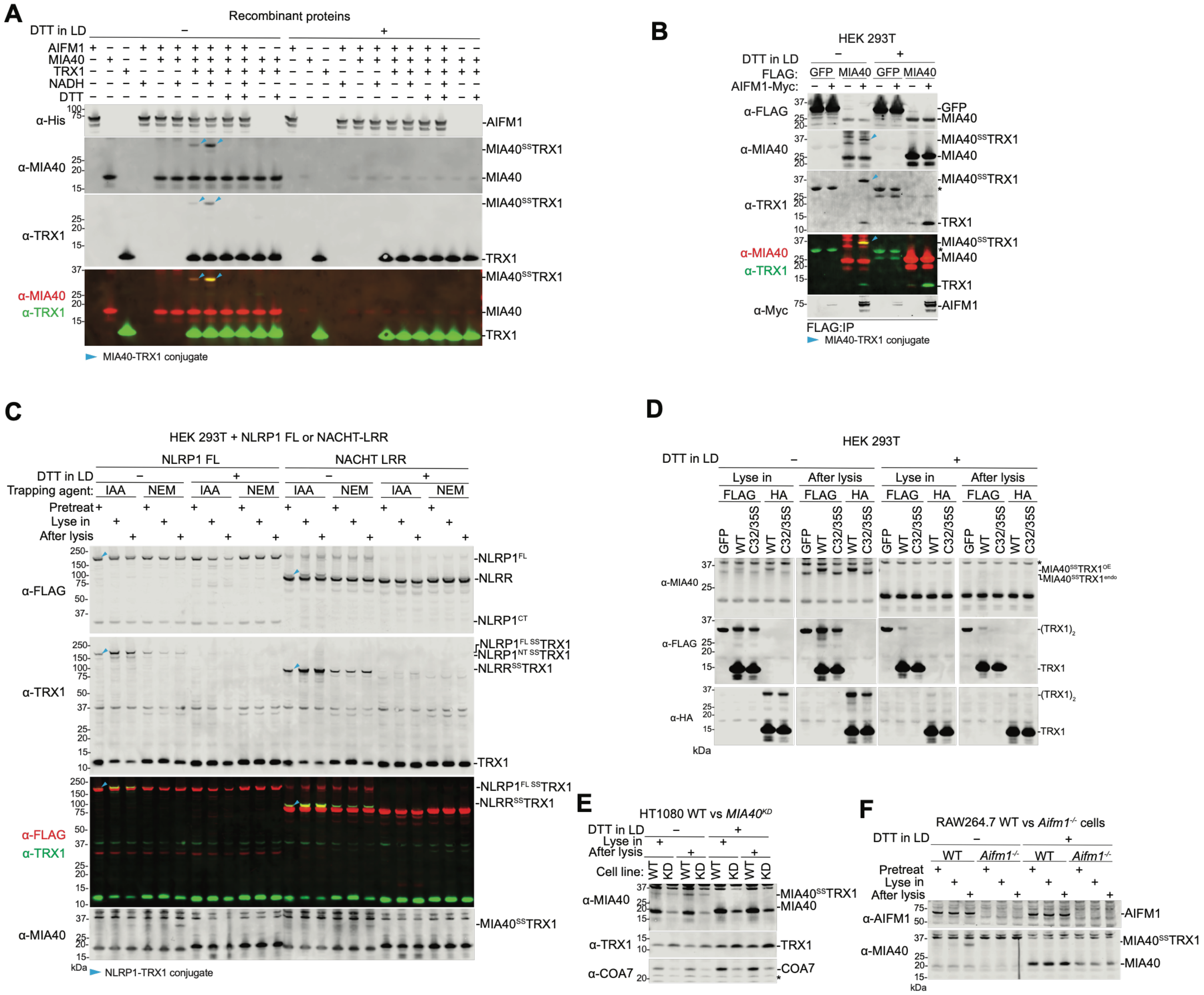

Figure S2. Characterization of the TRX1–MIA40 conjugate. **(A)** Recombinant AIFM1, MIA40, and TRX1 (Cys 62/69/73 mutated to Ser) were combined under the indicated DTT (10 mM) and NADH (100 µM) conditions, trapped with NEM (40 mM), and analyzed by immunoblotting under reducing or nonreducing conditions. Cyan arrowheads indicate MIA40–TRX1 conjugates. **(B–F)** FLAG-IPs from HEK 293T cells expressing the indicated proteins **(B–D)**, WT and MIA40-deficient HT1080 cells **(E)**, and WT and AIFM1-deficient RAW264.7 cells **(F)** were subjected to the indicated NEM (40 mM) and IAA (10 mM) thiol-trapping conditions and analyzed by immunoblotting under reducing or nonreducing conditions. Arrowheads indicate the MIA40–TRX1 or the NLRP1 FL– or NACHT LRR–TRX1 conjugates. Immunoblots are representative of three or more independent experiments. Asterisks denote non-specific bands.

bioRxiv preprint doi: https://doi.org/10.64898/2026.10.26.761918; this version posted October 28, 2026. The copyright holder for this preprint (which was not certified by peer review) is the author/funder, who has granted bioRxiv a license to display the preprint in perpetuity. It is made available under a CC-BY 4.0 International license.

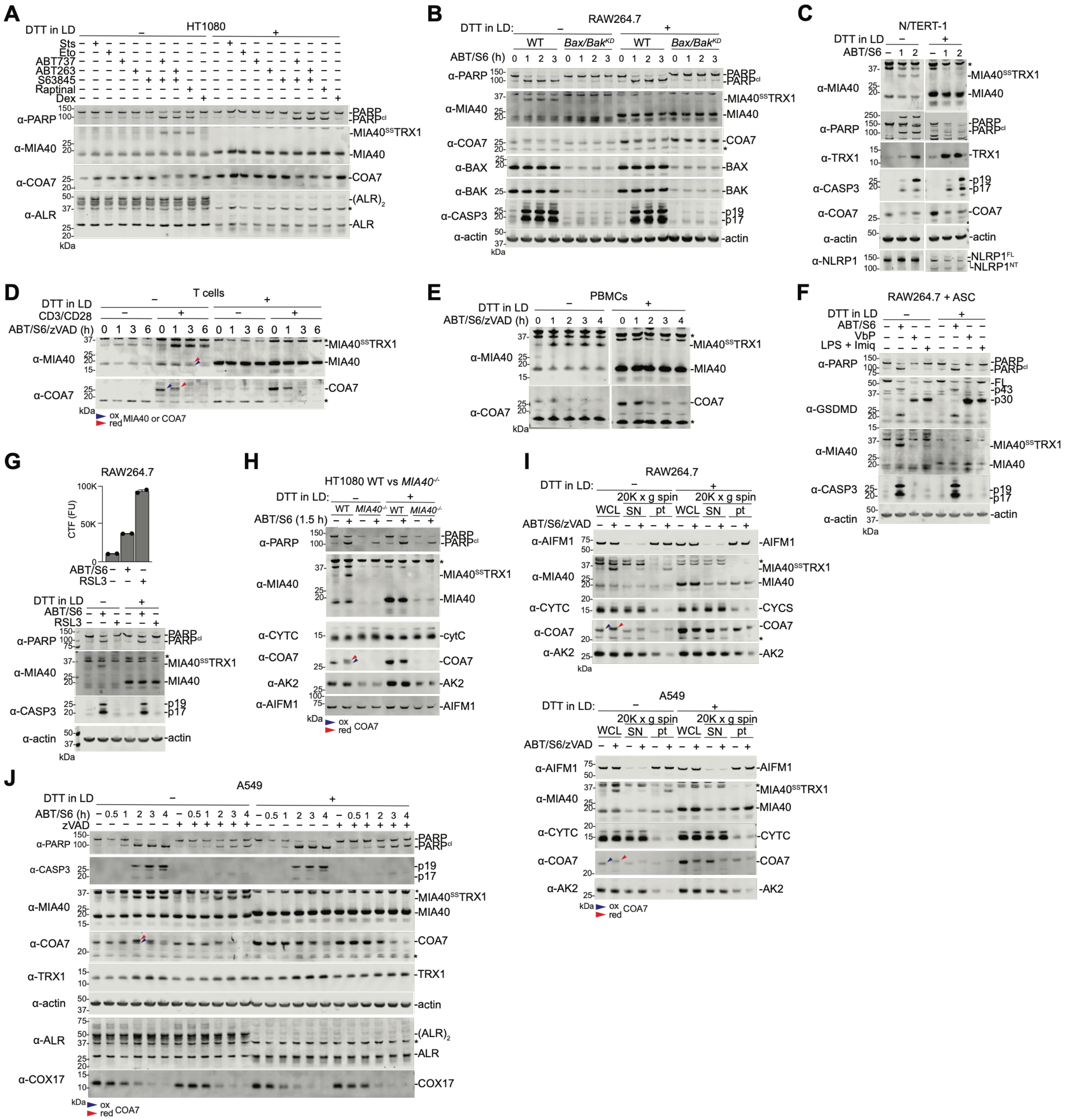

Figure S3. Intrinsic apoptosis induces MIA40-TRX1 conjugate formation. (A–J) The indicated cells were treated with the specified compounds for 2 h **(A,I)**, the indicated intervals **(B-E, H,J)**, or 3 h **(F,G)**. All cells were lysed in buffer containing 40 mM NEM, except in **C** was in 10 mM IAA, and samples were analyzed by immunoblotting under reducing or nonreducing conditions. In **(G)**, cell death was additionally measured by CytoTox-Fluor fluorescence. In **(I)**, lysates were separated into soluble and precipitate fractions by centrifugation. Compound concentrations: staurosporine (10 µM), etoposide (20 µM), ABT-737 (10 µM), ABT-263 (10 µM), S63845 (1 µM), raptinal (10 µM), dexamethasone (50 µM), zVAD (50 µM), VbP (10 µM), LPS (1 µg/mL), imiquimod (100 µM), and RSL3 (10 µM). Immunoblots are representative of three or more independent experiments. Asterisks denote non-specific bands.

bioRxiv preprint doi: https://doi.org/10.64898/2026.10.26.761918; this version posted October 28, 2026. The copyright holder for this preprint (which was not certified by peer review) is the author/funder, who has granted bioRxiv a license to display the preprint in perpetuity. It is made available under a CC-BY 4.0 International license.

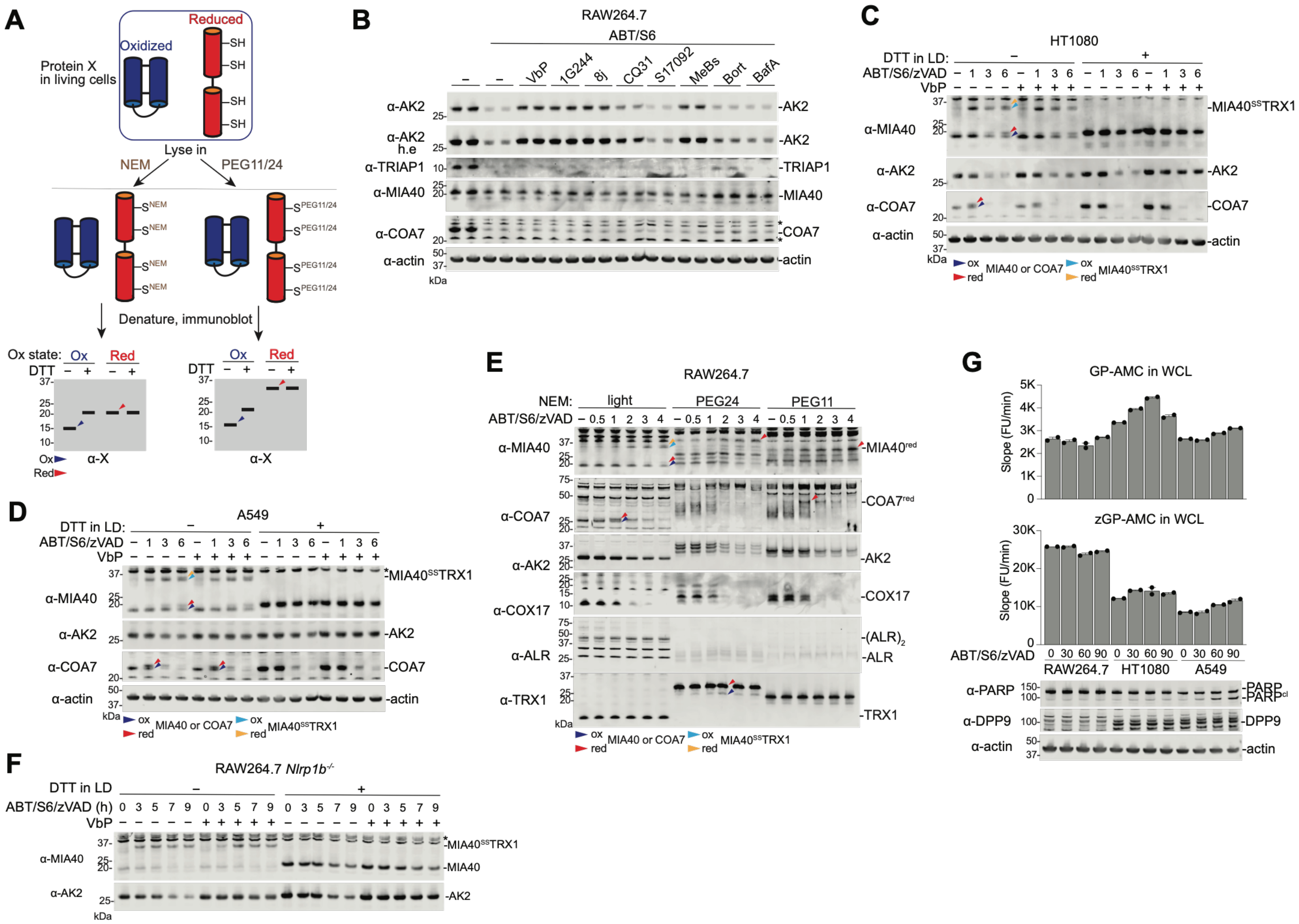

Figure S4. DPP8/9 inhibition blocks AK2 degradation after MOMP. **(A)** Schematic of NEM- and PEG-based thiol-labeling assays to distinguish oxidized and reduced protein species. **(B)** RAW264.7 cells were co-treated with zVAD, VX-765, and the indicated compounds for 3.5 h. **(C–F)** The indicated cells were treated with ABT/S6/zVAD for the indicated intervals ± VbP. **(E)** Redox-state analysis of the indicated proteins in RAW264.7 cells following ABT/S6/zVAD treatment for the indicated times. Arrowheads indicate the oxidized and reduced protein species and the corresponding MIA40–TRX1 conjugates, as indicated. All cells, except in **(B)** and the PEG-labeled samples in **(E)**, were lysed in buffer containing 40 mM NEM and analyzed by immunoblotting under reducing or nonreducing conditions. Samples in **(B)** were lysed without NEM and analyzed by immunoblotting only under reducing conditions. In **(E)**, the indicated samples were labeled with 15 mM PEG11 or PEG24 and analyzed under reducing conditions. **(G)** DPP8/9 and PREP activity in lysates from RAW264.7, HT-1080, and A549 cells following ABT/S6/zVAD treatment for the indicated times, measured using GP-AMC and zGP-AMC, respectively. Compound concentrations: ABT-263 (10 µM), S63845 (1 µM), VbP (10 µM), zVAD (50 µM), and VX-765 (50 µM). Compounds in **(B)** were used at 10 µM except bortezomib (500 nM) and bafilomycin A1 (100 nM). Immunoblots are representative of three or more independent experiments. Asterisks denote non-specific bands.

bioRxiv preprint doi: https://doi.org/10.64898/2026.10.26.761918; this version posted October 28, 2026. The copyright holder for this preprint (which was not certified by peer review) is the author/funder, who has granted bioRxiv a license to display the preprint in perpetuity. It is made available under a CC-BY 4.0 International license.

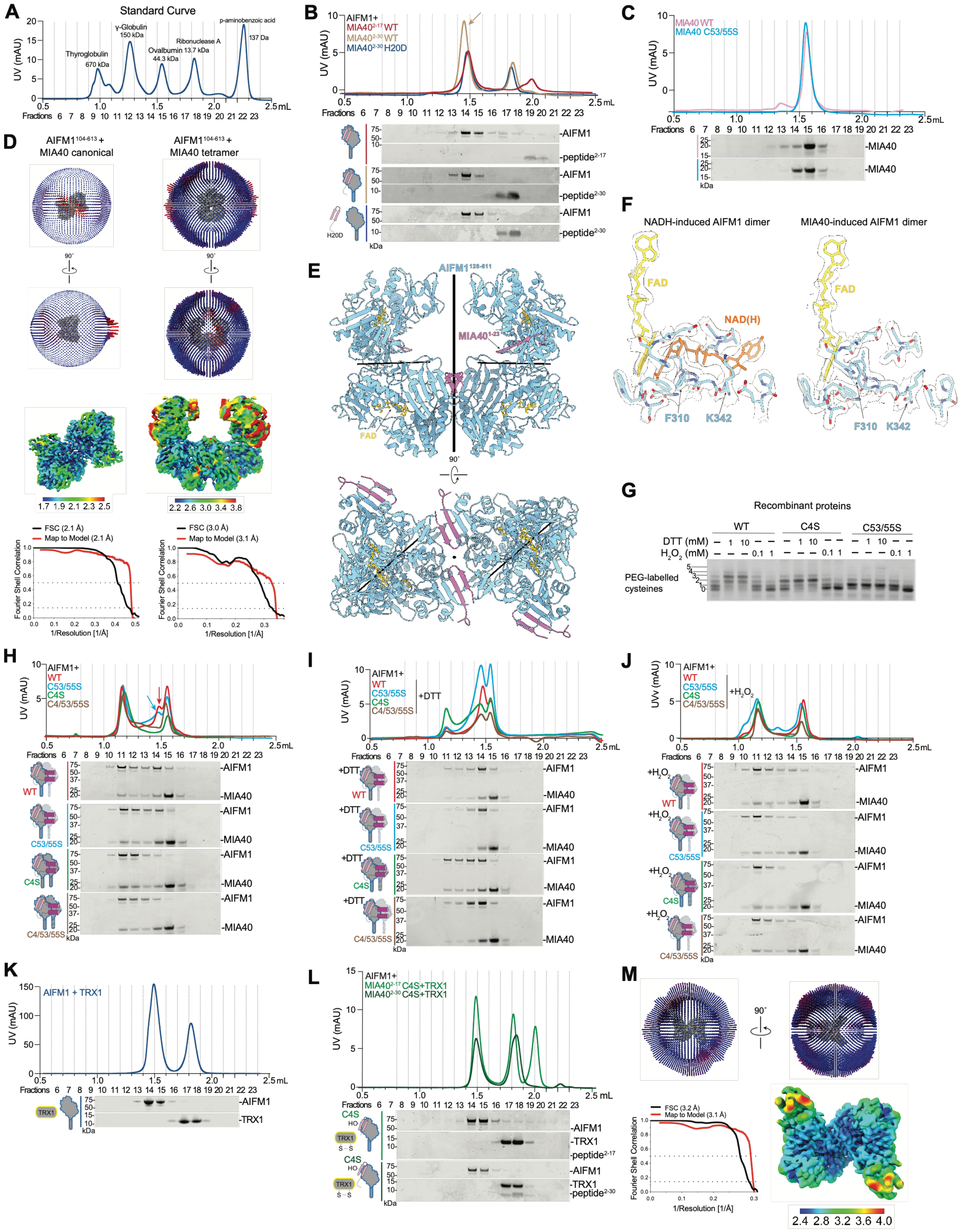

bioRxiv preprint doi: https://doi.org/10.64898/2026.10.26.761918; this version posted October 28, 2026. The copyright holder for this preprint (which was not certified by peer review) is the author/funder, who has granted bioRxiv a license to display the preprint in perpetuity. It is made available under a CC-BY 4.0 International license.

Figure S5. NADH-independent MIA40-driven AIFM1 dimerization. (**A**) SEC calibration using the indicated molecular weight standards. (**B,C,H-L**) SEC of the indicated proteins. DTT (1 mM) and H_2O_2 (100 µM) were added as indicated. The collected fractions were analyzed by Coomassie staining. Recombinant proteins were mixed at an AIFM1:MIA40:TRX1:peptide molar ratio of 1:2:4:10, with AIFM1 at 10 µM, except in (**K**) where AIFM1 is 35 uM. Arrow in (**B**) indicates binding of peptide to AIFM1 and arrow in (**H**) indicates the MIA40-bound AIFM1 monomer. TRX1 used in these experiments had Cys 62/69/73 mutated to Ser. In (**C**) the MIA40 WT chromatogram is also shown in Main Fig. 5A. (**D,M**) Cryo-EM particle orientation distributions, Fourier shell correlation curves, and cryo-EM maps of the canonical AIFM1–MIA40 dimer (left) and tetramer (right) reconstructions (**D**) and of the AIFM1–MIA40 peptide$^{2–17}$–TRX1 complex (**M**). (**E**) Cryo-EM structure of the AIFM1–MIA40 tetramer (PDB 38EC). Thick and thin black lines indicate global and local twofold symmetry axes, respectively. (**F**) Comparison of cryo-EM density at the AIFM1 nucleotide-binding pocket between the structure in **Figure 1H** (left) and the current structure (right). (**G**) PEG24-maleimide analysis of purified MIA40 before and after DTT or H_2O_2 treatment at the indicated concentrations. Chromatograms are representative of three or more independent experiments.

bioRxiv preprint doi: https://doi.org/10.64898/2026.10.26.761918; this version posted October 28, 2026. The copyright holder for this preprint (which was not certified by peer review) is the author/funder, who has granted bioRxiv a license to display the preprint in perpetuity. It is made available under a CC-BY 4.0 International license.

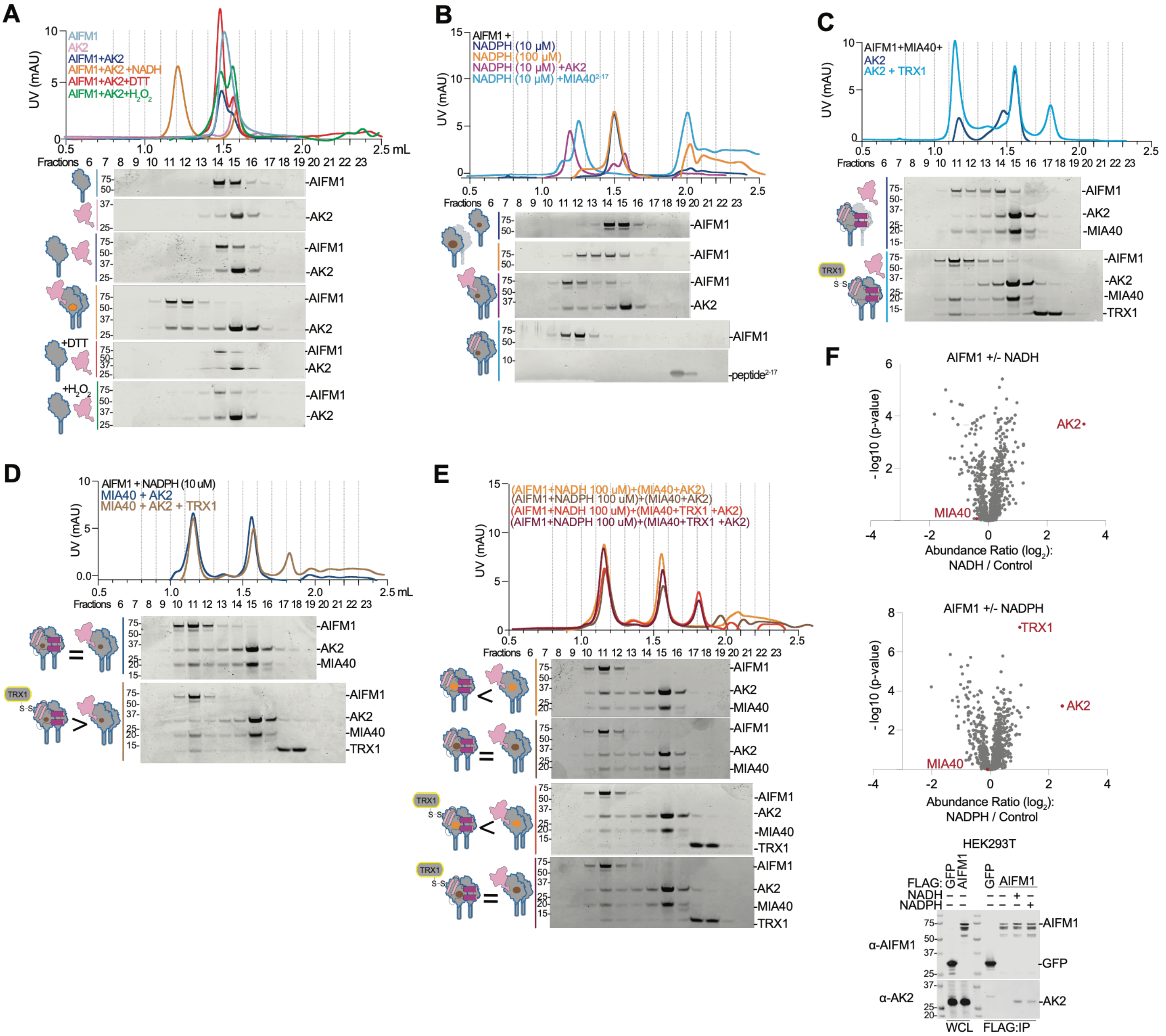

Figure S6. TRX1 increases stability of the MIA40-induced AIFM1 dimer. (A-E) SEC of recombinant AIFM1 with the indicated proteins, and peptides. DTT (1 mM), NADH (10 µM in **A** or as indicated), NADPH (10 or 100 µM as indicated) were added where indicated. Collected fractions were analyzed by Coomassie staining. Recombinant proteins were mixed at an AIFM1:MIA40:AK2:TRX1:MIA40 peptide molar ratio of 1:2:2:4:10, with AIFM1 at 10 µM. TRX1 Cys 62/69/73 Ser was used in these experiments. In **(A)**, the AIFM1 alone chromatogram/blot is also shown in **Figure 5A**. **(F)** Quantitative mass spectrometry of FLAG–AIFM1 immunoprecipitates in the absence or presence of NADH or NADPH (1 mM), with immunoblot validation shown at right.

bioRxiv preprint doi: https://doi.org/10.64898/2026.10.26.761918; this version posted October 28, 2026. The copyright holder for this preprint (which was not certified by peer review) is the author/funder, who has granted bioRxiv a license to display the preprint in perpetuity. It is made available under a CC-BY 4.0 International license.

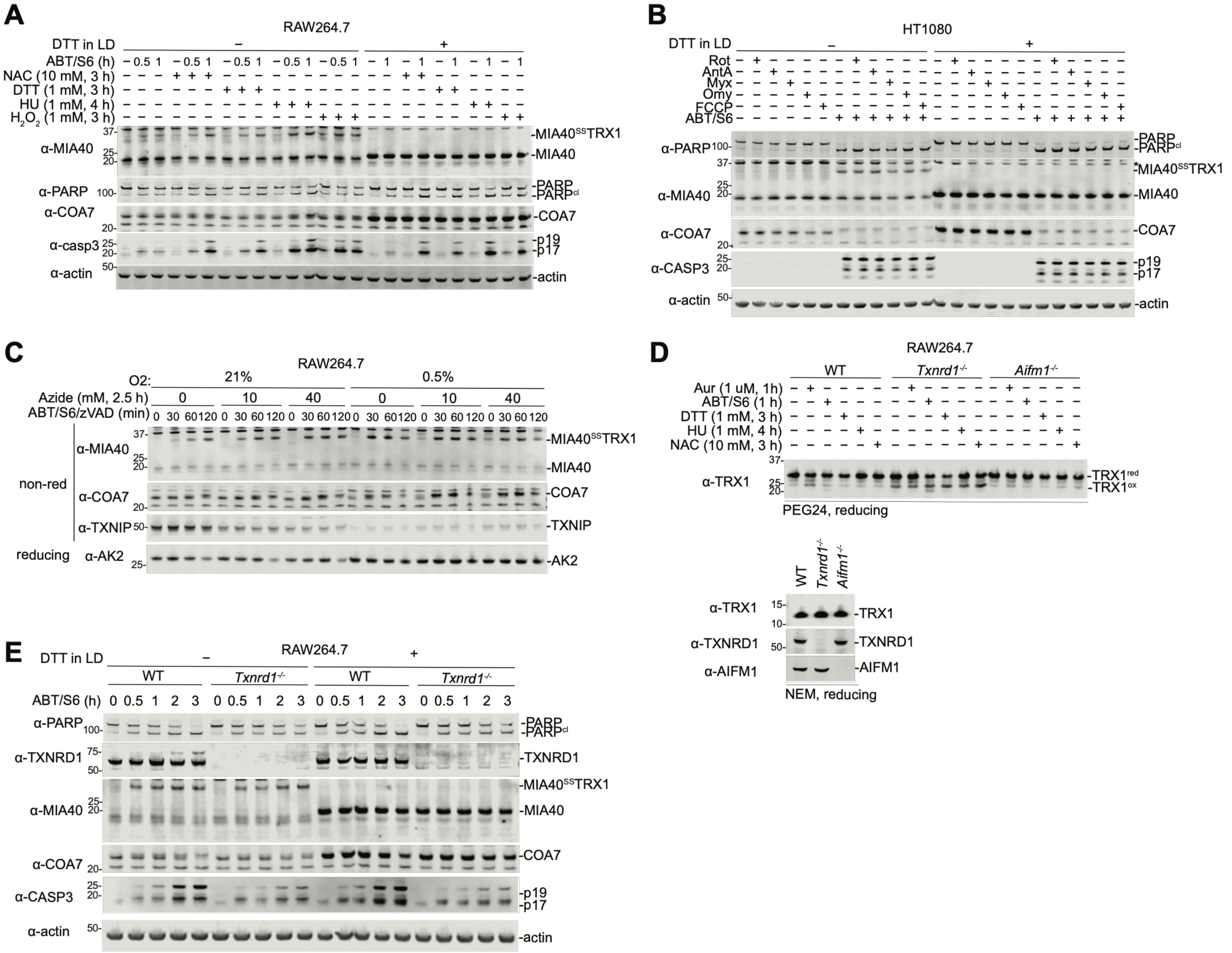

Figure S7. Redox perturbations do not alter conjugate formation. **(A–E)** The indicated cells were co-treated with ABT/S6 and the specified compounds for the indicated intervals. In **(C)**, cells were put in hypoxia for 30 h prior to treatment with azide and ABT/S6 as indicated. All cells were lysed in 40 mM NEM, except panel (**D**, top), which was lysed in NEM PEG24 (15 mM). Samples were analyzed by immunoblotting under reducing or nonreducing conditions as indicated. Immunoblots are representative of three or more independent experiments.
